# Comparing the Cost-Effectiveness of Orthognathic Surgery Treatment Between Orthodontics-First and Surgery-First Approaches in Thammasat University Hospital: A Retrospective Study

**DOI:** 10.3390/healthcare14121778

**Published:** 2026-06-19

**Authors:** Phetcharat Chatmongkhonkit, Narissaporn Chaiprakit, Arthessarat Sirisa-Ard, Siripatra Patchanee

**Affiliations:** 1 Division of Oral and Maxillofacial Surgery, Faculty of Dentistry, Thammasat University, Pathum Thani 12120, Thailand; phetploy@tu.ac.th (P.C.); cnarissp@tu.ac.th (N.C.); 2 Thammasat University Research Unit in Mineralized Tissue Reconstruction, Faculty of Dentistry, Thammasat University, Pathum Thani 12120, Thailand; sarthess@tu.ac.th; 3 Division of Orthodontics, Faculty of Dentistry, Thammasat University, Pathum Thani 12120, Thailand

**Keywords:** orthodontic-first approach (OFA), surgery-first approach (SFA), cost-effectiveness, quality of life, Orthognathic Quality of Life Questionnaire (OQLQ)

## Abstract

Objectives: This study aimed to evaluate and compare the cost-effectiveness of the surgery-first and orthodontic-first approaches in Thai patients with dentofacial deformities undergoing orthognathic surgery. Methods: This retrospective cohort study included 30 patients with dentofacial deformities, divided into the surgery-first approach (SFA, n = 15) and orthodontic-first approach (OFA, n = 15). All underwent double-jaw surgery without genioplasty performed by a single surgeon and orthodontist. Data collected included operative and orthodontic costs, operative time, total treatment and hospital costs, treatment duration, and length of hospital stay. Cost-effectiveness was assessed using the incremental cost-effectiveness ratio (ICER) and incremental time-effectiveness ratio (ITER), with effectiveness measured by quality of life via the Thai Orthognathic Quality of Life Questionnaire (OQLQ) before and at the time of debonding the orthodontic appliance. Results: Overall, the SFA demonstrated greater cost-effectiveness than the OFA. However, the SFA group incurred slightly higher hospital costs. There were no statistically significant differences between the two groups in operative cost, hospital cost, total cost, operative time, or length of hospital stay (*p* > 0.05). By contrast, orthodontic cost, orthodontic treatment duration, and total treatment duration were significantly lower in the SFA group compared with the OFA group (*p* < 0.05). Conclusions: Within limits, the SFA is a potentially beneficial alternative to OFA. Patients and clinicians may benefit from using SFA by experiencing shorter treatment duration, lower orthodontic treatment costs, and improvements in certain aspects of quality of life. Further studies with larger sample sizes and longitudinal data are necessary to establish the long-term effectiveness.

## 1. Introduction

Dentofacial deformities can significantly impair function and aesthetics, affecting mastication, facial harmony, psychosocial well-being, and overall quality of life (QOL) [1]. Managing these conditions typically involves orthognathic treatment—a combination of surgical and orthodontic interventions—to restore occlusal function and facial balance while minimizing treatment duration and cost. A patient-centered, cost-conscious approach is essential in modern healthcare settings, where value-based care is increasingly emphasized.

Orthognathic surgery remains the treatment of choice for correcting skeletal discrepancies. Two primary approaches are commonly employed: the orthodontic-first approach (OFA) and the surgery-first approach (SFA). OFA involves presurgical orthodontic treatment to decompensate the dentition before surgical correction. Although effective, this method is time-consuming and associated with potential complications during the presurgical phase, such as gingival recession, root resorption, and deterioration in facial aesthetics. By contrast, SFA omits the presurgical phase, allowing surgery to be performed first, followed by orthodontic treatment. SFA offers benefits such as reduced overall treatment time, earlier aesthetic improvement, and accelerated tooth movement due to the regional acceleratory phenomenon (RAP) [2]. However, it requires meticulous case selection and advanced expertise in treatment planning, as inadequate coordination between surgical and orthodontic phases can lead to suboptimal outcomes.

Cost-effectiveness has become a central consideration in healthcare decision-making. Cost-effectiveness analysis (CEA) is a standard method for comparing the cost and effectiveness of medical interventions. It quantifies the additional cost required to achieve one unit of health benefit when comparing two treatment options. Costs are typically expressed in monetary terms (e.g., Thai baht), while effectiveness may be measured in units such as life-years gained, deaths prevented, or QOL improvement [3]. In orthognathic surgery, QOL—often assessed using validated tools such as the 22-item Orthognathic Quality of Life Questionnaire (OQLQ)—can serve as a key indicator of treatment effectiveness [4].

While previous studies suggest that SFA can reduce overall treatment time compared with OFA, some have reported increased surgical complexity, long operative times, and surgical costs [5,6,7]. Although a shorter treatment duration may reduce orthodontic-related costs, its overall impact on cost-effectiveness remains unclear. However, only one previous study has directly compared the cost-effectiveness of SFA and OFA [5]. Furthermore, no such research has been conducted in the Thai context. Given this gap in the literature, the present study evaluates and compares the cost-effectiveness of the surgery-first and orthodontic-first approaches in Thai patients with dentofacial deformities undergoing orthognathic surgery. The evaluation will focus on total treatment duration, direct treatment costs, and changes in patient-reported QOL. This research aims to inform clinical decision-making and support the development of efficient, value-based care models in orthognathic treatment.

## 2. Materials and Methods

**Figure 1:**
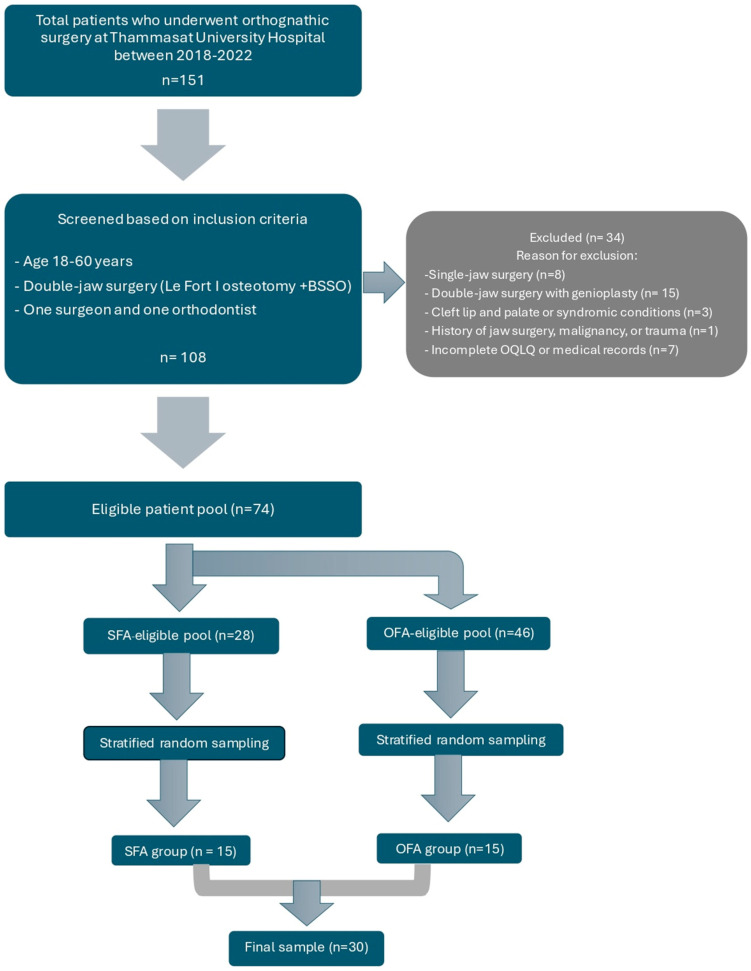
Flowchart of the patient selection process using stratified random sampling based on the treatment approach.

This retrospective cohort study included patients who underwent orthognathic surgery between 2018 and 2022. Clinical, surgical, and cost data were obtained from medical records. Follow-up data, including orthodontic treatment duration, orthodontic treatment costs, and postoperative OQLQ assessments, were collected through 2025. A total of 30 patients diagnosed with dentofacial deformities were divided equally into two groups: the surgery-first approach (SFA, n = 15) and the orthodontic-first approach (OFA, n = 15). The sample size was calculated using a standard cost-effectiveness sample size formula. All patients underwent double-jaw surgery without genioplasty, performed by a single oral and maxillofacial surgeon in collaboration with one orthodontist, at the Department of Oral and Maxillofacial Surgery and the Department of Orthodontics, Thammasat University Hospital, Thailand. This study was conducted at a single center. The patients received the orthognathic treatment at Thammasat Hospital (Figure 1). Ethical approval was granted by the Human Research Ethics Committee of Thammasat University (Science), Pathum Thani, Thailand (Project Code: 67DE054; Approval Date: 28 August 2024).

This study’s cost-effectiveness analysis comprises two key values: the incremental cost-effectiveness ratio (ICER) and the incremental time-effectiveness ratio (ITER). The ICER is defined as the difference in cost between SFA and OFA, divided by the difference in effectiveness of the two approaches. Similarly, the ITER is defined as the difference in treatment time between SFA and OFA, divided by the difference in effectiveness.

Costs (C) included in the ICER are limited to direct costs: intraoperative costs, total hospitalization costs, orthodontic treatment costs, and total treatment costs. All amounts are expressed in Thai baht. All costs were analyzed based on the actual historical values obtained from the hospital records. Inflation adjustments and discounting were not applied because both patient cohorts were treated contemporaneously within the same timeframe (2018–2025), and the institutional fixed tariffs regulated by the public university hospital remained static, eliminating potential temporal asymmetry between the groups.

Treatment time in the ITER comprises operation time (minutes), length of hospital stay (days), orthodontic treatment period (months), and total treatment duration (months).

Effectiveness (E) is measured by changes in QoL, as quantified by the OQLQ. OQLQ scores were collected at two time points: before treatment (Tx) and at the time of debonding the orthodontic device (Ty).

$$n=\frac{2{\left(z_{\alpha }+z_{\beta }\right)}^{2}[sd_{C}^{2}+(Ws\;d_{q}{)}^{2}-(2W\rho \;sd_{c}sd_{q})]}{({WQ-C)}^{2}}$$

**α** = Confidence (0.05);

**β** = Power (0.8);

***C*** = Expected point estimate for the difference in mean cost (412.74);

***n*** = Sample size per group;

***Q*** = Expected point estimate for the difference in mean effect (1.76);

ρ = The expected correlation of the difference in cost (C) and effect (Q);

***sd_c_*** = Expected SD for the cost in each treatment group (5.97);

***sd_q_*** = Expected SD for the effect in each treatment group (6.13);

***W*** = Maximum willingness to pay (1,250,000);

$z_{\alpha }$ = Z-statistic for the α error (1.96).

The expected SD for the cost, effect, and difference in mean cost comprises data from a previous study [5]. The expected correlation of the difference in cost and effect is a measure of the covariance of changes in effectiveness and changes in cost. It varies from −1 to 1, and the sample size reaches a minimum when ρ = 1 [8]. Using data from the previous study, we concluded that the average willingness to pay for life-saving treatment and subsequent quality-of-life improvement in Thailand for one year is 1,250,000 baht [9].

### 2.1. Inclusion and Exclusion Criteria

Participants were patients aged from 18 to 60 years diagnosed with dentofacial deformities, who underwent bimaxillary orthognathic surgery, and who were treated with orthognathic treatment at Thammasat University Hospital from 2018 to 2025. The maxilla underwent a single-piece Le Fort I osteotomy, while the mandible underwent a bilateral sagittal split osteotomy (BSSO). Patients were split into two groups: the OFA group, who underwent pre-surgical orthodontic preparation and regular post-surgical orthodontic adjustments, and the SFA group, who underwent surgery without preoperative orthodontic therapy. The OQLQ was only completed by patients before and after treatment. Patients with congenital conditions or syndromes associated with maxillofacial deformities (such as cleft lip and palate), mental illness, trauma-related maxillofacial changes, cancer, temporomandibular joint dysfunction, prior orthognathic surgery, or systemic disorders that might impair their quality of life were excluded.

The ICER and ITER were the two components of the cost-effectiveness analysis. The ICER is calculated by dividing the various costs (C) of SFA and OFA by their respective efficacy (E).

The ICER and ITER equations are shown below:

$${ICER}_{mean}=\frac{mean\left(C_{1}\right)-mean(C_{2})}{mean\left(E_{1}\right)-mean(E_{2})}$$

C1 = Cost of treatment in SFA;

C2 = Cost of treatment in OFA;

E1 = Effectiveness of SFA (the difference in the OQLQ scores between Tx and Ty of SFA);

E2 = Effectiveness of OFA (the difference in OQLQ scores between Tx and Ty of OFA).

$${ITER}_{mean}=\frac{mean\left(T_{1}\right)-mean(T_{2})}{mean\left(E_{1}\right)-mean(E_{2})}$$

T1 = Time of treatment in SFA;

T2 = Time of treatment in OFA;

E1 = Effectiveness of SFA (the difference in the OQLQ scores between Tx and Ty of SFA);

E2 = Effectiveness of OFA (the difference in OQLQ scores between Tx and Ty of OFA).

The mean of ICER is reported in terms of the ICERs of intraoperative cost, total cost of hospitalization, orthodontic treatment cost, and total cost of treatment. The mean of ITER is reported in terms of the ITERs of operation time, length of hospital stay, orthodontic treatment time, and total treatment time.

### 2.2. Statistical Analysis

Data normality was assessed using the Shapiro–Wilk test, visual inspection of histograms, and evaluation of skewness. As several variables were not normally distributed and the sample size was small, non-parametric tests were used. Accordingly, comparisons between groups were performed using the Mann–Whitney *U* test. Gender distribution between groups was compared using Pearson’s chi-square test. Age, OQLQ scores before treatment and at debonding, operation cost, hospital cost, operation time, length of hospital stay, orthodontic treatment time, total treatment time, orthodontic cost, and total treatment cost were compared between the groups using the Mann–Whitney *U* test. A *p*-value less than 0.05 was considered statistically significant.

### 2.3. Cost-Effectiveness Analysis

The incremental time-effectiveness ratio (ITER and the incremental cost-effectiveness ratio (ICER comprise the study’s cost-effectiveness analysis. The ICER is calculated by dividing the cost difference (C) between OFA and SFA by the effectiveness difference (E) between the two groups. In the same way, the ITER shows the ratio of the two treatments’ differences in efficacy to their differences in treatment duration.

In this study, costs (C) include only direct expenses, specifically intraoperative costs, total hospitalization costs, orthodontic treatment costs, and the overall cost of treatment. All costs are expressed in monetary terms (Thai baht).

Treatment time comprises several components: operation time (measured in minutes), length of hospital stays (in days), orthodontic treatment duration (in months), and total treatment time (in months).

Effectiveness (E) is assessed based on changes in QOL, as measured using the OQLQ. OQLQ scores were recorded before treatment (Tx) and again at the time of debonding the orthodontic appliance (Ty). The difference in OQLQ scores between these two time points is the effectiveness parameter used to calculate the ICER and ITER.

According to data interpretation, an intervention is considered “dominated” if its costs are higher and its effectiveness is lower than that of the comparator. However, an intervention is considered “dominant” if its costs are lower and its effectiveness is higher than that of the comparator. The most frequent situation, known as “trade-off,” is when a novel approach improves clinical outcomes at a comparatively high expense. Making decisions in trade-off situations is based on one’s willingness to pay [10].

The hospital bank’s data were used to gather cost information, including intraoperative costs (baht), the total cost of hospitalization (baht), orthodontic treatment cost (baht), total cost of treatment (baht), operation time (minutes), length of hospital stay (days), orthodontic treatment time (months), and total treatment time (months). Information about quality of life before and after surgery was collected from the OQLQ, where some of the information already exists. The OQLQ is a preoperative and postoperative treatment regimen in the dentistry clinic at Thammasat University Hospital. Permission was granted for all information requested from the hospital before the data were collected. To obtain permission from the hospital, a formal letter was sent to the director of Thammasat Hospital. Only researchers can access these data.

The 22-item Orthognathic Quality of Life Questionnaire (OQLQ)

Twenty-two items on the OQLQ (Thai version) were rated on a four-point scale, with 1 indicating “it bothers you a little,” 4 indicating “it bothers you a lot,” and 2 and 3 indicating “it bothers you somewhere in the middle.” NA indicates that the statement does not apply to you or bothers you. The total OQLQ scores ranged from 0 to 88. A lower score indicates a higher quality of life, whereas a higher score indicates a lower quality. The 22 questions are divided into four categories: oral function (items 2–6, scoring 0–20), knowledge of dentofacial esthetics (items 8, 9, 12, and 13, scoring 0–16), social aspects of dentofacial deformity (items 15–22, scoring 0–32), and facial esthetics (items 1, 7, 10, 11, and 14, scoring 0–20). The OQLQ scores were collected before treatment (Tx) and after debonding of the orthodontic device (Ty). The Thai version of OQLQ was used in a previous study, “Comparing quality of life in orthognathic surgery patients between orthodontic-first and surgery-first approaches” [4,11,12].

## 3. Results

In total, 30 patients were divided into two groups: SFA and OFA. There were 12 males and 18 females. The ratio of males to females between the two groups was compared using Pearson’s chi-square test, which yielded a *p*-value of 0.273, indicating no significant difference between the groups. As several variables were not normally distributed and the sample size was small, non-parametric tests were used. Between-group comparisons were performed using the Mann–Whitney *U* test. The mean age of patients in the OFA group was 27.27 ± 1.66 years, while that of the SFA group was 24.87 ± 1.67 years (*p* = 0.227). The sample included patients with skeletal Class II and III deformities, with more than 93% presenting with Class III deformity. The mean pretreatment OQLQ scores in the OFA and SFA groups were 51.93 ± 4.72 and 51.27 ± 4.93, respectively, with no statistically significant difference (*p* = 0.923) (Table 1).

At the debonding stage, the mean OQLQ scores for the OFA and SFA groups were 21.53 ± 2.93 and 18.73 ± 3.23, respectively. The median OQLQ scores were 24 (18) for the OFA group and 16 (20) for the SFA group (*p* = 0.442). The operative costs in the OFA group were slightly higher than in the SFA group; however, the difference was not statistically significant (*p* =0.290). Conversely, hospital costs in the OFA group were slightly lower than those in the SFA group, possibly due to the shorter hospital stay in the OFA group. Nevertheless, there were no statistically significant differences in either hospital costs (*p* = 0.548) or length of hospital stay (*p* = 0.66) between the two groups. The operative time also showed no significant difference between groups (*p* = 0.803).

By contrast, the orthodontic treatment duration was significantly longer in the OFA group, with patients requiring approximately 14.2 additional months of treatment compared with those in the SFA group (*p* = 0.001). This finding corresponds with the markedly longer overall treatment duration recorded in the OFA group. Notably, the orthodontic component of treatment was substantially more expensive in the OFA group, exceeding the SFA group by approximately 10,600 baht (*p* = 0.005). Although the OFA group also incurred a higher total treatment cost—by roughly 7700 baht—the magnitude of this difference did not reach statistical significance (*p* = 0.237). Together, these results highlight the economic implications associated with prolonged treatment timelines in the OFA (Table 2).

Changes in OQLQ scores were evaluated for the OFA and SFA groups. The mean ± SD improvements in total OQLQ scores were 30.40 ± 14.00 for the OFA group and 32.53 ± 18.89 for the SFA group. Although the SFA group demonstrated a greater mean improvement in total OQLQ scores, the difference between groups was not statistically significant (*p* = 0.771). No statistically significant differences were observed across OQLQ subdomains. Overall, increases in OQLQ scores reflected improved quality of life following treatment in both groups, with comparable outcomes between the two surgical approaches (Table 3).

During the surgical phase, the SFA was associated with greater cost efficiency in operative expenses, with an ICER indicating savings of 2425.63 baht per additional ∆OQLQ point gained compared with the OFA group. Conversely, the ICER for hospital costs showed that the SFA group required 1364.18 baht more per additional ∆OQLQ point gained. The ITER analysis further indicated that the SFA group required 0.47 min of operative time and 0.06 days of hospital stay per additional ∆OQLQ point gained.

**Figure 2:**
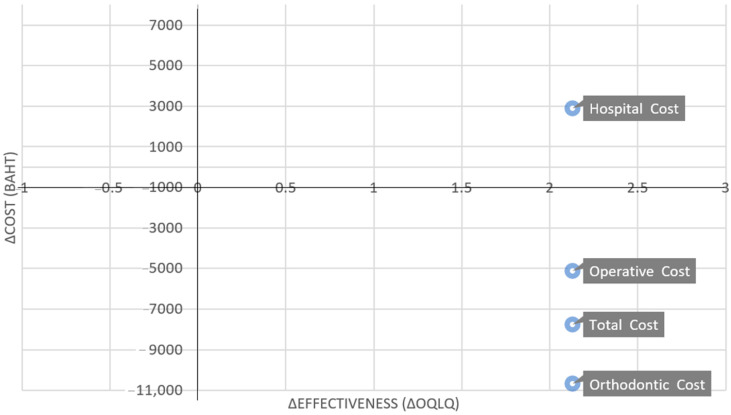
Cost-effectiveness plane comparing SFA and OFA based on incremental cost and effectiveness (∆OQLQ).

In the orthodontic phase, the SFA group exhibited lower orthodontic treatment costs, with an ICER indicating savings of 5007.37 baht per additional ∆OQLQ point gained relative to the OFA group. The ITER results were consistent with these findings, showing that the SFA group required 6.67 fewer months of orthodontic treatment time per additional ∆OQLQ point gained. Considering the entire treatment period, SFA appeared more cost-effective, with an ICER indicating savings of 3643.19 baht per additional ∆OQLQ point gained and an ITER demonstrating a reduction of 6.67 months in total treatment duration compared with the OFA group (Table 4 and Figure 2).

Overall, SFA was associated with lower operative and orthodontic costs and a shorter treatment duration compared with OFA. However, hospital costs were slightly higher in the SFA group. Although SFA demonstrated lower mean costs and slightly greater effectiveness than OFA, the difference in effectiveness was not statistically significant.

## 4. Discussion

Orthognathic treatment can be performed using either SFA or OFA and is the treatment of choice for correcting dentofacial deformities. Both approaches have advantages and disadvantages. This study compared the clinical outcomes, treatment duration, quality of life (OQLQ), and cost-effectiveness between SFA and OFA in orthognathic treatment to aid in decision-making for proper treatment in each patient, considering cost-effectiveness.

Baseline demographic characteristics—including sex distribution, age, and pretreatment OQLQ scores—showed no significant differences between groups, indicating appropriate comparability. Most participants presented with skeletal Class III deformities.

In the surgical phase, Hu et al. found that while SFA was associated with a shorter total treatment duration than OFA, their SFA group’s intraoperative time was noticeably longer [5]. Conversely, the current study did not show statistically significant differences in operative time or cost between the SFA and OFA groups, which is in line with the results of our earlier study [13]. This apparent difference could be the result of variations in operative procedures, surgical expertise, and case selection. According to Jeong et al., the impact of surgical factors on operative efficiency and cost appears to be reduced when orthognathic procedures are carried out by a single experienced surgeon using standardized techniques and a well-defined surgical plan [14].

However, it should be noted that a learning curve is associated with SFA, and cases involving severe vertical or transverse discrepancies necessitate careful thought and meticulous treatment planning. To minimize intraoperative occlusal instability, surgical wafers are still necessary to precisely replicate planned maxillary and/or mandibular movements based on surgical treatment objectives and model surgery [15]. The predictability and accuracy of SFA have significantly improved with recent developments in cone-beam computed tomography, virtual surgical planning, and digital wafer fabrication [16,17], which may help to explain the correlated operative outcomes seen in this study. There were no significant variations between the two groups regarding postoperative recovery or length of hospital stay, which is consistent with earlier research [5]. In the same way, there was no significant difference in hospital expenses between the SFA and OFA groups.

The surgical phase was relatively short, and the healing period was comparable between groups. As a result, differences in these phases had minimal impact on the overall treatment timeline, which was primarily determined by the orthodontic treatment duration.

Regarding orthodontic treatment related to orthognathic surgery, the current study found that patients in the OFA group needed approximately 14.2 more months of treatment than those in the SFA group. The shorter orthodontic treatment duration in the SFA group may be related to eliminating the presurgical orthodontic decompensation phase and the potential benefits of the RAP following surgery. This finding is in line with previous studies showing variations in the length of orthodontic treatment between the two approaches [14,18,19,20].

Notably, the length of orthodontic treatment depends on several variables, such as the degree of skeletal discrepancies, the complexity of malocclusion, and the type of surgery performed [14,21,22]. Therefore, rather than indicating the intrinsic superiority of one treatment protocol over another, the longer orthodontic treatment duration found in the OFA group in this study was associated with a longer overall treatment duration. Additionally, there is presently no agreement on the best time to receive orthodontic treatment before or after surgery [23].

Biological reactions during recovery, such as accelerated alveolar bone turnover and improved blood flow—a phenomenon known as the regional acceleratory phenomenon (RAP)—may impact post-surgical orthodontic tooth movement [2]. Active orthodontic tooth movement begins during this biologically advantageous postoperative phase in SFA, enabling more efficient use of RAP. However, patients treated with OFA usually experience significant orthodontic decompensation before surgery, resulting in comparatively less orthodontic tooth movement after the procedure. The shorter orthodontic treatment time seen in the SFA group, particularly during the early stages of postoperative orthodontic treatment, may be partially explained by this difference [15,24].

According to earlier research, postsurgical orthodontics should be initiated directly after surgery, as well as two or four to six weeks later, after the maxillomandibular fixation is removed and sufficient mouth opening is achieved [25,26]. In this study, orthodontic treatment began about two weeks following surgery; however, neither the length of orthodontic treatment nor the overall treatment time showed any significant differences between the two groups. Systematic reviews have similarly shown that total treatment time tends to be shorter in SFA than in OFA, although considerable variability exists, with reported mean differences in postoperative orthodontic treatment time ranging from approximately 6 to 12 months [14,18,19,25,26,27].

The current study found that the OFA group had considerably longer treatment durations during the orthodontic phase of orthognathic treatment, which was associated with higher orthodontic-related expenses. In particular, the OFA group’s orthodontic component of treatment was substantially greater than that of the SFA group, by almost 10,600 baht. The OFA group also had a greater total treatment cost—roughly 7700 baht—but this difference was not statistically significant. These results imply that longer orthodontic treatment and more frequent visits may affect cost distribution and patient time commitment, even though there are no statistically significant differences. Consistent with previous reports [20], the SFA group required fewer orthodontic appointments than the OFA group, which may partly explain the observed differences in orthodontic costs and resource utilization.

In the SFA and OFA groups, quality-of-life outcomes improved from pretreatment to the debonding stage. There were no statistically significant changes between groups or OQLQ subdomains, despite the SFA group’s somewhat greater improvement in overall OQLQ scores. These results are comparable to other research and show that both treatment modalities are linked to similar patient-reported quality-of-life outcomes after orthognathic treatment [4,28]. There is currently insufficient evidence to suggest that skeletal classification significantly affects OQLQ outcomes. Several studies have found no significant correlation between gender and postoperative OQLQ scores [1,29,30,31], but others suggest potential gender-related differences [32].

The results of this study are generally consistent with previous research [5] comparing SFA and OFA protocols. During the surgical phase, SFA was associated with lower operative costs per additional ∆OQLQ point, although hospital-related expenditures were slightly higher, and differences in operative time and length of hospital stay were minimal, similar to prior findings [1]. More pronounced differences were observed during the orthodontic phase, with the SFA group saving approximately 5000 baht per additional ∆OQLQ point and shortening treatment duration by an average of 6.7 months. When considering the entire treatment course, ICER and ITER analyses suggest that SFA and OFA differ in total cost and treatment efficiency, while providing comparable improvements in patient quality of life.

This study has several limitations. First, the retrospective design may have introduced selection bias. No matching method, including propensity score matching, was performed, and multivariable adjustment was not conducted due to the relatively small sample size. Therefore, residual confounding from measured and unmeasured variables cannot be completely excluded. In addition, important confounding factors that may have influenced treatment outcomes and costs were not available for analysis. Second, the sample size was calculated based on estimates from a previous study; however, the variability observed in some outcomes was greater than anticipated, which may have limited the statistical power to detect differences between groups. Third, the direct costs were analyzed using the actual costs recorded at the time of treatment and were not adjusted for inflation. Although patients in both groups were treated during similar time periods, changes in the value of money over time may have influenced the absolute cost estimates. However, the relatively small sample size may still limit the robustness and generalizability of the findings. Given that healthcare cost and clinical outcome data are often skewed, future studies with larger sample sizes are recommended to confirm these results.

## 5. Conclusions

Within the aforementioned limitations, the current study suggests that SFA is a potentially beneficial alternative to OFA. Patients and clinicians may benefit from using SFA by experiencing shorter treatment duration, lower orthodontic treatment costs, and improvement in certain aspects of quality of life. Further studies with larger sample sizes and longitudinal data are necessary to establish the long-term effectiveness of SFA and gain insight into its implications for clinical practice.

## Figures and Tables

**Table 1:** Baseline demographic and clinical characteristics of patients in the SFA and OFA groups.

	OFA	SFA	Total	*p*-Value
Gender (n,%)				
Male	7 (46.67%)	5 (33.33%)	12 (40%)	0.273 (Pearson’s chi-square test)
Female	8 (53.33%)	10 (66.67%)	18 (60%)	
Age (years)				
Mean +/− SD	27.27 ± 1.66	24.87 ± 1.67		
Median [IQR]	27 (9)	23 (4)		0.227 (Mann–Whitney *U* test)
Type of deformity				
Skeletal II (n,%)	1 (3.33%)	1 (3.33%)	2 (6.67%)	
Skeletal III (n,%)	14 (96.67%)	14 (96.67%)	28 (93.33%)	
Average OQLQ score before treatment				
Mean +/− SD	51.93 ± 4.72	51.27 ± 4.93		
Median [IQR]	52 (22)	43 (33)		0.648 (Mann–Whitney *U* test)

n, number of samples; SD, standard deviation. The significance level is 0.05.

**Table 2:** Comparison of clinical outcomes, treatment duration, and costs between OFA and SFA groups at debonding stage analyzed using Mann–Whitney *U* test.

Outcome	Statistic	OFA	SFA	*p*-Value (Mann–Whitney *U* Test)
n		15	15	
OQLQ score at debonding	Mean +/− SD	21.53 ± 2.93	18.73 ± 3.23	
	Median [IQR]	24 (18)	16 (20)	0.442
Operation cost (material costs, billed minutes; baht)	Mean +/− SD	75,934 ± 3188.22	70,767.40 ± 3660.17	
	Median [IQR]	74,680 (19,524)	65,198 (21,936)	0.290
Hospital costs (operative costs and pharmacy/hospital costs; baht)	Mean +/− SD	95,537.40 ± 3637.68	98,443.10 ± 4119.21	
	Median [IQR]	97,321 (25,309)	103,585 (25,190)	0.548
Operation time (minutes)	Mean +/− SD	328.67 ± 24.62	329 ± 20.29	
	Median [IQR]	300 (160)	315 (117)	0.803
Length of stay (day)	Mean +/− SD	4.27 ± 0.18	4.40 ± 0.24	
	Median [IQR]	4 (1)	4 (1)	0.660
Orthodontic time (months)	Mean +/− SD	44.47 ± 2.11	30.27 ± 2.91	
	Median [IQR]	43 (14)	25 (16)	0.001
Total treatment time (months)	Mean +/− SD	44.47 ± 2.11	30.27 ± 2.91	
	Median [IQR]	43 (14)	25 (16)	0.001
Orthodontic cost (baht)	Mean +/− SD	70,153.07 ± 2165.80	59,487.37 ± 2592.67	
	Median [IQR]	68,125 (6475)	58,920 (14,500)	0.005
Total cost of treatment (baht)	Mean +/− SD	165,690.47 ± 4265.68	157,930.46 ± 5329.92	
	Median [IQR]	164,862 (22,644)	157,698 (24,624)	0.237

Note: n, number of samples; SD, standard deviation. The significance level is 0.05.

**Table 3:** Comparison of quality-of-life improvement (OQLQ) between OFA and SFA groups analyzed using Mann–Whitney *U* test.

∆OQLQ Tx-Ty			
Domain	OFA Mean Difference +/− SD (Median [IQR])	SFA Mean Difference +/− SD (Median [IQR])	*p*-Value (Mann–Whitney *U* test)
Social (0–32)	9.33 ± 1.55 (9 [7])	11.46 ± 2.28 (11 [13])	0.787
Esthetic (0–20)	9.13 ± 1.07 (7 [9])	7.80 ± 1.67 (8 [11])	0.454
Function (0–20)	5.73 ± 1.30 (4 [7])	6.53 ± 1.34 (6 [7])	0.454
Awareness (0–16)	6.20 ± 1.00 (7 [6])	6.73 ± 1.31 (7 [10])	0.803
Total (0–88)	30.40 ± 14.00 (26 [26])	32.53 ± 18.89 (31 [24])	0.771

Tx, time before treatment; Ty, time after debonding of the orthodontic device. The significance level is 0.05.

**Table 4:** Incremental cost-effectiveness and time-effectiveness analysis (ICER and ITER) of SFA versus OFA groups.

Variables	Mean ± SD	Between-Treatment Increment	Mean ± SD Difference OQLQ Score Between Tx and Ty	Between-Treatment Increment	ICER or ITER	95% CI
Cost-effectiveness of operative cost						
SFA	70,767.40 ± 3660.17	−5166.6	32.53 ± 18.89	2.13	−2425.63 (baht/∆OQLQ)	[−14,071.2,182,93.84]
OFA	75934 ± 3188.22		30.40 ± 14.00			
Time-effectiveness of operative time						
SFA	329 ± 20.29	1	32.53 ± 18.89	2.13	0.47 (min/∆OQLQ)	
OFA	328.67 ± 24.62		30.40 ± 14.00			
Cost-effectiveness of hospital cost						
SFA	98,443.10 ± 4119.21	2905.7	32.53 ± 18.89	2.13	1364.18 (baht/∆OQLQ)	[−25,358.66,27,621.13]
OFA	95,537.40 ± 3637.68		30.40 ± 14.00			
Time-effectiveness of length of hospital stay						
SFA	4.40 ± 0.24	0.13	32.53 ± 18.89	2.13	0.06 (day/∆OQLQ)	
OFA	4.27 ± 0.18		30.40 ± 14.00			
Cost-effectiveness of orthodontic cost						
SFA	59,487.37 ± 2592.67	−10,665.7	32.53 ± 18.89	2.13	−5007.37 (baht/∆OQLQ)	[−25,358.66,27,621.13]
OFA	70,153.07 ± 2165.80		30.40 ± 14.00			
Time-effectiveness of orthodontic time						
SFA	30.27 ± 2.91	−14.2	32.53 ± 18.89	2.13	−6.67 (month/∆OQLQ)	
OFA	44.47 ± 2.11		30.40 ± 14.00			
Cost-effectiveness of total treatment cost						
SFA	157,930.46 ± 5329.92	−7760.01	32.53 ± 18.89	2.13	−3643.19 (baht/∆OQLQ)	[−21,873.68,24,505.77]
OFA	165,690.47 ± 4265.68		30.40 ± 14.00			
Time-effectiveness of total treatment time						
SFA	30.27 ± 2.91	−14.2	32.53 ± 18.89	2.13	−6.67 (month/∆OQLQ)	
OFA	44.47 ± 2.11		30.40 ± 14.00			

Tx, time before treatment; Ty, time after debonding of the orthodontic device; 95%CI, 95% Confidence Interval. ICER, incremental cost-effectiveness ratio; ITER, incremental time-effectiveness ratio; SD, standard deviation. The significance level is 0.05.

## Data Availability

The data presented in this study are available upon request from the corresponding author due to privacy and ethical restrictions, as the dataset contains sensitive clinical information with potential risk of de-identification.

## Abbreviations

The following abbreviations are used in this manuscript:

SFA	Surgery-first approach
OFA	Orthodontic-first approach
CEA	Cost-effectiveness analysis
ICER	Incremental cost-effectiveness ratio
ITER	Incremental time-effectiveness ratio
C	Cost
E	Effectiveness
T	Time
OQLQ	Orthognathic Quality of Life Questionnaire
QOL	Quality of life
SD	Standard deviation
BSSO	Bilateral sagittal split osteotomy
WTP	Willingness to pay
